# ERRATUM

**DOI:** 10.1111/os.12731

**Published:** 2020-08-28

In [1], the information of all authors were incorrect in the published version.

The below information,

Yongzheng Bao, MD^1^, Xueren Thong, MM^1^, Wengang Zhu, MM^1^, Yu Chen, BS^1^, Longze Zhou, MM^1^, Xiangheng Dai, MM^1^, Junjian Liao, MM^1^, Zhong Li, MM^3^, Konghe Hu, MD^1^, Kangsheng Bei, BS^1^, Yinghui Xiong, BS^1^, Yongyu Hu, BS^1^, Qinfu Zhao, BS^2^, Zhouxing Zhu; BS^2^, Yanli Yu, BS^1^, Qiang Wu, MD^1^, Xinhua Xi, MM^1^.

^*1*^ *Department of Spinal Surgery, Nanfang Hospital, Southern Medical University, Guangzhou and* ^*2*^ *Department of Orthopedic Surgery, Lechang People's Hospital, Shaoguan and* ^*3*^ *Department of Spine Surgery, Jingmen Second People's Hospital Jingmen, China*.

should be corrected as:

Yongzheng Bao MD^1^, Xueren Zhong MM^1^, Wengang Zhu MM^1^, Yu Chen BS^1^, Longze Zhou MM^1^, Xiangheng Dai MM^2^, Junjian Liao MM^1^, Zhong Li MM^3^, Konghe Hu MD^1^, Kangsheng Bei BS^1^, Yinghui Xiong BS^1^, Yongyu Hu BS^1^, Qinfu Zhao BS^4^, Zhouxing Zhu BS^4^, Yanli Yu BS^1^, Qiang Wu MD^1^^※^, Xinhua Xi MM^1^^※^

^1^Department of Spine Surgery, Yuebei People's Hospital Affiliated to Shantou University Medical College, Shaoguan City, Guangdong Province, China, 512026.

^2^Department of Spinal Surgery, Nanfang Hospital, Southern Medical University, Guangzhou City, Guangdong Province, China, 510515.

^3^Department of Spine Surgery, Jingmen Second People's Hospital, Jingmen City, Hubei Province, China, 448000.

^4^Department of Orthopedic Surgery, Lechang People's Hospital, Shaoguan City, Guangdong Province, China, 512200.

Co‐first authors: Yongzheng Bao, Xueren Zhong.

Corresponding author: Qiang Wu, E‐mail: sgwuqiang@sina.com; Xinhua Xi, 56249042@qq.com

The publisher apologizes for these errors and any convenience these may have caused.
